# Association of long-term use of dipeptidyl peptidase-4 inhibitors with the risk of diabetic retinopathy in patients with diabetes mellitus: a real-world evidence study

**DOI:** 10.3389/fphar.2025.1518545

**Published:** 2025-04-16

**Authors:** Yu-Ching Li, Yu-Hsiang Kuan, Yih Yang, Shuo-Yan Gau, Kun-Yu Su, Tung-Han Tsai, Kuang-Hua Huang, Chien-Ying Lee

**Affiliations:** ^1^ Department of Public Health, China Medical University, Taichung, Taiwan; ^2^ Division of Family Medicine, Yuan Rung Hospital, Changhua, Taiwan; ^3^ Department of Pharmacology, Chung Shan Medical University, Taichung, Taiwan; ^4^ Department of Pharmacy, Chung Shan Medical University Hospital, Taichung, Taiwan; ^5^ Department of Surgery, E-Da Hospital, I-Shou University, Kaohsiung, Taiwan; ^6^ Department of Business Administration, National Taiwan University, Taipei, Taiwan; ^7^ School of Medicine, Chung Shan Medical University, Taichung, Taiwan; ^8^ Department of Health Services Administration, China Medical University, Taichung, Taiwan

**Keywords:** diabetic retinopathy, dipeptidyl peptidase-4 inhibitors, diabetes mellitus, cumulative defined daily dose, real-world evidence

## Abstract

**Background:**

In this study, we investigated the association of long-term use of a dipeptidyl peptidase-4 inhibitor (DPP-4i) with the risk of diabetic retinopathy (DR) in patients with diabetes mellitus (DM).

**Methods:**

This study was a secondary analysis based on the nationwide database from 2008 to 2022 in Taiwan. Patients with new-onset DM who were treated with either a DPP-4i or sulfonylurea from 2009 to 2017 were included in this study. Patients who received a DPP-4i were included in the case group and further divided on the basis of the cumulative defined daily dose (cDDD) as follows: ≤90, 91–180, 181–300, and >300 cDDD. Propensity score matching was performed to select patients who used a sulfonylurea, and these patients were assigned to the control group. With adjustment for sex, age, income, urbanization level, comorbidities, and other anti-diabetic agents, the Cox proportional hazard model was used to estimate the risk of DR associated with DPP-4i use over the 5-year follow-up.

**Results:**

There were 83,503 patients with DPP-4i use and 167,006 patients with sulfonylurea use after matching. Compared with patients with sulfonylurea use, patients with DPP-4i use at ≤90 cDDD had a hazard ratio (HR) of 1.43 (95% confidence interval [CI] = 1.38–1.49) for DR development, whereas those with DPP-4i use at 91–180, 181–300 or >300 cDDD had HRs of 1.66 (95% CI: 1.59–1.74), 1.82 (95% CI: 1.74–1.90), and 2.32 (95% CI: 1.91–2.82) for DR development, respectively. Of the different DPP-4is, linagliptin at ≤90 or 181–300 was associated with the highest risk of DR. Significant differences were discovered at ≤90, 91–181, and 181–300 cDDD in the risk of DR between patients using Saxagliptin versus sitagliptin. Vildagliptin at ≤90 or 91–180 cDDD was associated with an increased risk of DR, but not at 181–300 cDDD.

**Conclusion:**

In patients with DM, DPP-4i at ≤90, 91–180, 181–300 and >300 cDDD was linked to an increased risk of DR over the 5-year follow-up. Sitagliptin at cDDD 181–300 was associated with the greatest DR risk. The potential for DPP-4i to accelerate DR progression should be considered.

## Highlights

• This study found that long-term use of DPP-4 inhibitors in patients with type 2 diabetes is linked to an increased risk of developing diabetic retinopathy (DR). The risk was higher with greater exposure to these medications, particularly for sitagliptin.
• Among the different DPP-4 inhibitors, sitagliptin was associated with the highest risk. These findings suggest that careful monitoring may be needed for diabetic patients using DPP-4 inhibitors to manage their blood sugar.

## Introduction

Diabetic retinopathy (DR) is a common and severe microvascular complication of diabetes mellitus (DM) and is a leading cause of vision loss among adults in developed countries (Sabanayagam et al., 2019). Multiple factors are involved in the development of DR. Hyperglycemia, advanced glycation end products (AGEs), hyperglycemia-induced oxidative stress, and low-grade inflammation may lead to DR development (Li et al., 2018). The progression of DR to the proliferative phase is primarily driven by retinal inflammation, retinal neovascularization, and endothelial activation (Joussen et al., 2004; Tang et al., 1994; Chahed et al., 2010). Multiple factors contribute to DR development. Specifically, one study reported that prolonged hyperglycemia leads to the accumulation of AGEs in retinal cells, which plays a significant role in DR development (Zong et al., 2011). DR progression is primarily driven by the duration of DM and the resulting hyperglycemia. A longer duration of DM is associated with a higher risk of DR (Leley et al., 2021). DR is a chronic and progressive microvascular complication of DM and categorized into two stages: the initial stage of nonproliferative diabetic retinopathy and the advanced stage of proliferative diabetic retinopathy. This classification is based on observable pathological changes in the eyes and the presence of retinal neovascularization (Mahajan et al., 2019).

Glucagon-like peptide-1 (GLP-1) is rapidly degraded by dipeptidyl peptidase-4 (DPP-4). DPP-4 also cleaves several other substrates, such as stromal cell-derived factor-1 alpha (SDF-1α), which may play a major role in the development of diabetic retina. SDF-1α level is elevated in proliferative DR, and SDF-1α promotes angiogenesis (Butler et al., 2005). Ischemic tissues of animals with SDF-1 α overexpression exhibit increased neovascularization (Deshane et al., 2007). Whether a decrease in SDF-1α level contributes to early vasoregression is unclear. The degradation products of active GLP-1, namely, GLP-1 (9–37) amide and GLP-1 (9–36) amide, are believed to suppress the excessive production of mitochondrial reactive oxygen species (Nishikawa et al., 2000; Giacco et al., 2015). Recent studies have indicated that DPP-4 inhibitors (DPP-4is) may worsen DR (Kim et al., 2018)-(14). For example, DPP-4is may worsen DR by increasing retinal vascular permeability, raising concerns about their safety in patients with DR (Lee et al., 2016). DPP-4is prevent the degradation of SDF-1α, thus leading to increases in its active concentration (Lambeir et al., 2001; Fujita et al., 2014). Due to the neovascular effects of SDF-1, the additional increased SDF-1 may exert adverse effects in terms of causing proliferation and damage that are similar to the pathological processes involved in DR development (Butler et al., 2005). Therefore, inhibiting DPP-4 may diminish the vascular protection provided by the cleavage products of GLP-1. Given these conflicting results, predicting the overall impact of DPP-4 inhibition on diabetic microvascular damage is challenging.

In a previous study, short-term treatment with saxagliptin resulted in a decrease in retinal capillary flow in the microcirculation in patients with DM (Ott et al., 2014). Additionally, other small-scale studies have found that DPP-4is are associated with slow DR progression (Chung et al., 2016; Kolaczynski et al., 2016). However, in trials and a meta-analysis, DPP-4is were found to be associated with an increased risk of DR (Kim et al., 2018; Tang et al., 2018). Thus, the current evidence indicates that the association between DPP-4i treatment and the risk of DR in patients with DM is still under debate. In this study, we conducted a large-scale nationwide analysis to determine whether DPP-4i use is associated with an increased risk of DR. We also used a nationwide database, namely, the National Health Insurance Research Database (NHIRD) of Taiwan, to assess whether DPP-4i use dose-dependently increases the risk of DR in patients with DM.

## Materials and methods

### Data sources

In this study, we used a secondary database linked to the NHIRD that covers the period from 2008 to 2022. This database is maintained by the Health and Welfare Data Science Center (HWDC) of the Ministry of Health and Welfare of Taiwan. The NHIRD contains the information of all beneficiaries covered by the National Health Insurance program of Taiwan. This insurance program is a government-run, single-payer national social insurance program that was established in 1995. The database also contains the health insurance claims of 99% of the entire population of Taiwan (approximately 23 million people). Disease diagnoses are recorded in accordance with *International Classification of Diseases, Ninth Revision, Clinical Modification* (*ICD-9-CM*) and *International Classification of Diseases, Tenth Revision, Clinical Modification* (*ICD-10-CM*). Typically, the NHIRD provides real-world data that are used to support clinical decision-making and healthcare policymaking (Hsieh et al., 2019; Lai et al., 2020). In this study, we used data from the NHIRD to evaluate the risk of DR in patients with type 2 DM who were treated with DPP-4is.

### Ethics

This study was conducted in accordance with the Declaration of Helsinki and used data from the NHIRD, which is maintained by the HWDC. This database provides scrambled random identification numbers for insurants to ensure their privacy. The study protocol was approved by the Central Regional Research Ethics Committee of China Medical University, Taichung, Taiwan (approval no. CRREC-109-011). To ensure the privacy of patients, all data were anonymized. Because the database contains only deidentified data, the requirement for informed consent was waived.

### Study participants

**FIGURE 1:**
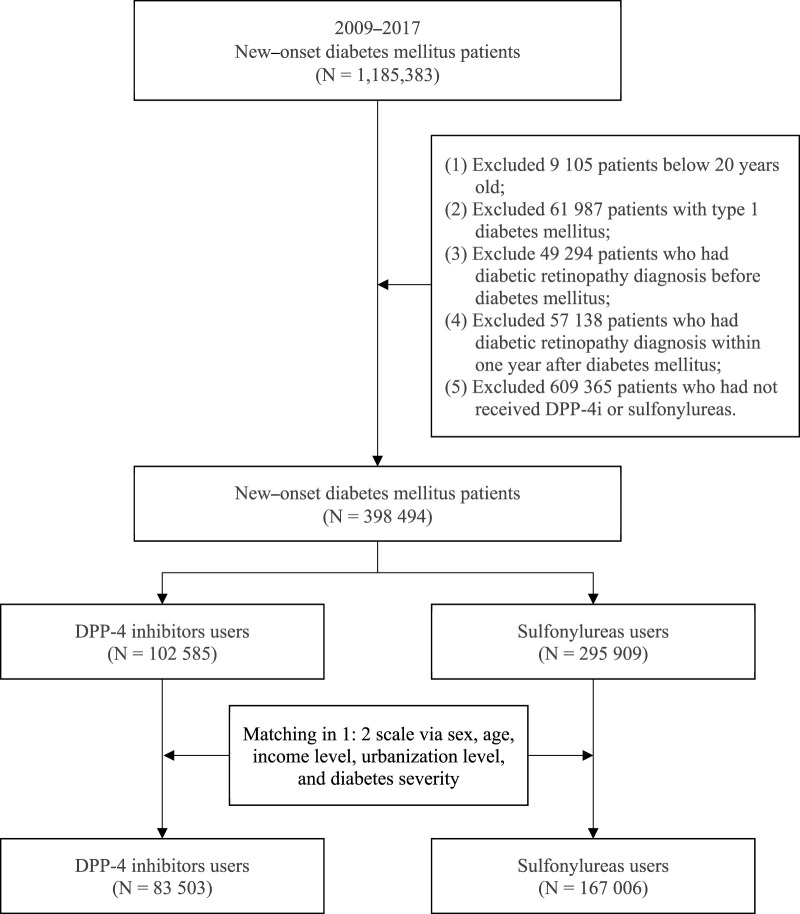
Patient selection process.

This study enrolled patients aged 20 years or older who received a diagnosis of type 2 DM (new-onset DM) from 2009 to 2017. DM (*ICD-9-CM* code 250 and *ICD-10-CM* code E08-E13) was defined as at least three records of outpatient diagnoses in a year. To reduce bias, the following patients were excluded: (Sabanayagam et al., 2019): patients with type 1 DM, (Li et al., 2018), patients who received a diagnosis of DR before DM development or a diagnosis of DR in the first year after DM development, and (Joussen et al., 2004) patients who had not received a DPP-4i or sulfonylurea. After excluding the aforementioned patients, a total of 398,494 patients with new-onset DM who were treated with either a DPP-4i or sulfonylurea from 2009 to 2017 were included in this study. We divided patients into two groups according to the diabetes medication within the first receiving after a diagnosis of DM: a case group (patients treated with a DPP-4i) and a comparison group (patients treated with a sulfonylurea). Medication classification in this study was based on the Anatomical Therapeutic Chemical (ATC) system. Sulfonylureas, used as the comparison group, were categorized under the ATC code A10BB. Four types of DPP-4 inhibitors were analyzed: linagliptin (A10BH05), saxagliptin (A10BH03), sitagliptin (A10BH01), and vildagliptin (A10BH02). Furthermore, to reduce potential confounding caused by unbalanced covariates in nonexperimental settings, we used the propensity score matching (PSM) method to address differences in baseline characteristics. The patients with DPP-4i and sulfonylurea use were matched at a 1:2 matching ratio. The variables used for matching were sex, age, insured salary, urbanization, diabetes complications severity index (DCSI), and the year of inclusion in the study. After matching, the study comprised 83,503 patients with DPP-4i use and 167,006 patients with sulfonylurea use. Figure 1 depicts the flowchart of patient selection.

### Study design

This cohort study with 5-year follow-up was conducted to investigate the risk of DR in patients with DM who were prescribed a DPP-4i in comparison with those who were prescribed a sulfonylurea. The first prescription date of a DPP-4i was the observation start date in patients with DPP-4i use. The first prescription date of a sulfonylurea was the observation start date in patients with sulfonylurea use. The included patients were regarded as having been continuously exposed to a DPP-4i or sulfonylurea during the study period. All patients were followed up for 5 years from the observation start date until their death, the use of another study drug (DPP-4i or sulfonylurea), the incidence of DR, or the end of the follow-up period, whichever occurred first. To measure the intake of DPP-4is, we used the defined daily dose (DDD), which is a standard measure of drug use and exposure. According to the World Health Organization, the DDD is the assumed average maintenance dose per day for adults. However, the DDD does not necessarily reflect the recommended or prescribed daily dose. Therefore, we calculated the cumulative DDD (cDDD) of DDP-4is within the follow-up period. The patients enrolled in this study were further divided on the basis of their cDDD of a DDP-4i as follows: ≤90, 91–180, 181–300, and >300 cDDD.

### Main outcome and covariates

DR was defined as three or more records with the *ICD-9-CM* code 362.0, 362.02, 362.07 or *ICD-10-CM* code H35.0, E08.31–E08.35, E09.31–E09.35, E11.31–E11.35, E13.31–E13.35 within 1 year. The control variables of the present study contained sex, age, income level, urbanization, DCSI, related comorbidities, and other anti-diabetic agents. Comorbidities were defined per the outpatient visit and admission record 1 year before the DM diagnosis, including obesity (*ICD-9-CM* code 278 and *ICD-10-CM* code E66), anxiety (*ICD-9-CM* code 300.0 and *ICD-10-CM* code F41), depression (*ICD-9-CM* code 296.2, 296.3 and *ICD-10-CM* code F32, F33), hypothyroidism (*ICD-9-CM* code 244.9 and *ICD-10-CM* code E03.9), hyperthyroidism (*ICD-9-CM* code 242.9 and *ICD-10-CM* code E05), migraine (*ICD-9-CM* code 346.9 and *ICD-10-CM* code G43, G44), chronic kidney disease (CKD, *ICD-9-CM* code 585 and *ICD-10-CM* code N18), hypertension (*ICD-9-CM* code 401–405 and *ICD-10-CM* code I10–I13, I15), and hyperlipidemia (*ICD-9-CM* code 272 and *ICD-10-CM* code E78). Other anti-diabetic agents were defined per the medication record within the follow-up period, including meglitinides, metformin, a-glucosidase inhibitors, thiazolidinediones (TZD), and insulins.

### Statistical analysis

All statistical analyses were conducted using SAS version 9.4 (SAS Institute, Cary, NC, United States). A *P* value of <0.05 indicated statistical significance. A chi-square test was conducted to determine the differences in baseline characteristics between the DPP-4i and sulfonylurea groups. With adjustment for sex, age, income level, urbanization, comorbidities, and other anti-diabetic agents, the Cox proportional hazard model was used to estimate the differences in the risk of DR between the patients with DPP-4i use and those with sulfonylurea use. Hazard ratios (HRs) with 95% confidence intervals (CIs) were calculated to determine the risk of DR associated with the use of a DPP-4i at ≤90, 91–180, 181–300, and >300 cDDD. We also employed the Kaplan–Meier method to identify differences in the cumulative incidence of DR between patients using DPP-4i and sulfonylureas. We further conducted subgroup analyses to investigate the risk of DR in patients using different DPP-4is. To explore potential effect modification by key comorbidities and concomitant antidiabetic medications, we performed additional subgroup analyses stratified by the presence of selected comorbidities (including obesity, anxiety, depression, hypothyroidism, hyperthyroidism, migraine, chronic kidney disease, hypertension, and hyperlipidemia) and the use of other antidiabetic medications (such as meglitinides, metformin, α-glucosidase inhibitors, TZD, and insulin).

## Results

Table 1 lists the baseline characteristics of the enrolled patients. The average age was 55.16 ± 12.74 years. Moreover, 34.09%, 15.96%, 16.41%, 13.30%, and 20.23% of the patients were aged 20–49, 50–54, 55–59, 60–64, and ≥65 years, respectively. In addition, 58.51% of the patients were male. After matching, no significant differences in sex, age, income level, urbanization, and DCSI between the patients with DPP-4i use and those with sulfonylurea use (*P* > 0.05). In the patients treated with a DPP-4i, 150 patients (0.18%) had obesity, 146 patients (0.17%) had anxiety, 178 patients (0.21%) had depression, 70 patients (0.08%) had hypothyroidism, and 110 patients (0.13%) had hyperthyroidism, 36 patients (0.04%) had migraine, 379 patients (0.45%) had CKD, 6 908 patients (8.27%) had hypertension, and 3 000 patients (3.59%) had hyperlipidemia. Regarding other anti-diabetic agents use, 6 644 patients (7.96%) took meglitinides, 67,052 patients (80.30%) took metformin, 11,687 patients (14.00%) took α-glucosidase inhibitors, 8 827 patients (10.57%) took TZD, and 9 181 patients (10.99) took insulins. Significant differences were discovered in the distribution of these comorbidities and other anti-diabetic agents use between the patients with DPP-4i use and those with sulfonylurea use (*P* < 0.05).

**TABLE 1:** Baseline characteristics of patients treated with DPP-4is and sulfonylureas.

Variables	Total		Sulfonylurea		DPP-4i		p-value
	N	%	N	%	N	%	
Total	250,509	100.00	167,006	100.00	83,503	100.00	
Sex^a^							0.722
Female	103,928	41.49	69,244	41.46	34,684	41.54	
Male	146,581	58.51	97,762	58.54	48,819	58.46	
Age (year)^a^							0.772
20–49	85,407	34.09	57,077	34.18	28,330	33.93	
50–54	39,993	15.96	26,666	15.97	13,327	15.96	
55–59	41,110	16.41	27,357	16.38	13,753	16.47	
66–64	33,325	13.30	22,194	13.29	11,131	13.33	
≥65	50,674	20.23	33,712	20.19	16,962	20.31	
Mean ± SD	55.16 ± 12.74		55.09 ± 12.53		55.30 ± 13.16		
Income level (NTD)^a^ ^,^ ^b^							0.920
≤19,200	58,972	23.54	39,362	23.57	19,610	23.48	
19,201–22,800	83,270	33.24	55,442	33.20	27,828	33.33	
22,801–36,300	46,175	18.43	30,781	18.43	15,394	18.44	
≥36,301	62,092	24.79	41,421	24.80	20,671	24.75	
Urbanization^a^							0.881
Level 1	67,961	27.13	45,337	27.15	22,624	27.09	
Level 2	78,539	31.35	52,403	31.38	26,136	31.30	
Level 3	43,775	17.47	29,195	17.48	14,580	17.46	
Level 4	34,583	13.81	23,063	13.81	11,520	13.80	
Level 5	5,075	2.03	3,357	2.01	1,718	2.06	
Level 6	10,330	4.12	6,880	4.12	3,450	4.13	
Level 7	10,246	4.09	6,771	4.05	3,475	4.16	
DCSI^a^ ^,^ ^b^							0.855
0	177,216	70.74	118,225	70.79	58,991	70.65	
1	40,356	16.11	26,869	16.09	13,487	16.15	
2	20,952	8.36	13,921	8.34	7,031	8.42	
≥3	11,985	4.78	7,991	4.78	3,994	4.78	
Comorbidities							
Obesity							<0.001
No	250,193	99.87	166,840	99.90	83,353	99.82	
Yes	316	0.13	166	0.10	150	0.18	
Anxiety							0.003
No	250,151	99.86	166,794	99.87	83,357	99.83	
Yes	358	0.14	212	0.13	146	0.17	
Depression							<0.001
No	250,123	99.85	166,798	99.88	83,325	99.79	
Yes	386	0.15	208	0.12	178	0.21	
Hypothyroidism							<0.001
No	250,355	99.94	166,922	99.95	83,433	99.92	
Yes	154	0.06	84	0.05	70	0.08	
Hyperthyroidism							<0.001
No	250,274	99.91	166,881	99.93	83,393	99.87	
Yes	235	0.09	125	0.07	110	0.13	
Migraine							0.021
No	250,430	99.97	166,963	99.97	83,467	99.96	
Yes	79	0.03	43	0.03	36	0.04	
CKD^b^							<0.001
No	249,851	99.74	166,727	99.83	83,124	99.55	
Yes	658	0.26	279	0.17	379	0.45	
Hypertension							<0.001
No	234,065	93.44	157,470	94.29	76,595	91.73	
Yes	16,444	6.56	9,536	5.71	6,908	8.27	
Hyperlipidemia							<0.001
No	243,543	97.22	163,040	97.63	80,503	96.41	
Yes	6,966	2.78	3,966	2.37	3,000	3.59	
Other anti-diabetic agents							
Meglitinides							<0.001
No	237,764	94.91	160,905	96.35	76,859	92.04	
Yes	12,745	5.09	6,101	3.65	6,644	7.96	
Metformin							<0.001
No	42,618	17.01	26,167	15.67	16,451	19.70	
Yes	207,891	82.99	140,839	84.33	67,052	80.30	
α-glucosidase inhibitors							<0.001
No	224,360	89.56	152,544	91.34	71,816	86.00	
Yes	26,149	10.44	14,462	8.66	11,687	14.00	
TZD^b^							<0.001
No	228,902	91.37	154,226	92.35	74,676	89.43	
Yes	21,607	8.63	12,780	7.65	8,827	10.57	
Insulins							<0.001
No	231,895	92.57	157,573	94.35	74,322	89.01	
Yes	18,614	7.43	9,433	5.65	9,181	10.99	

^a^ Variables for propensity score matching.

^b^ Abbreviations: NTD, New Taiwan dollars (1 NTD, 0.03 USD); DCSI, diabetes complications severity index; CKD, chronic kidney disease; TZD, thiazolidinediones.

**FIGURE 2:**
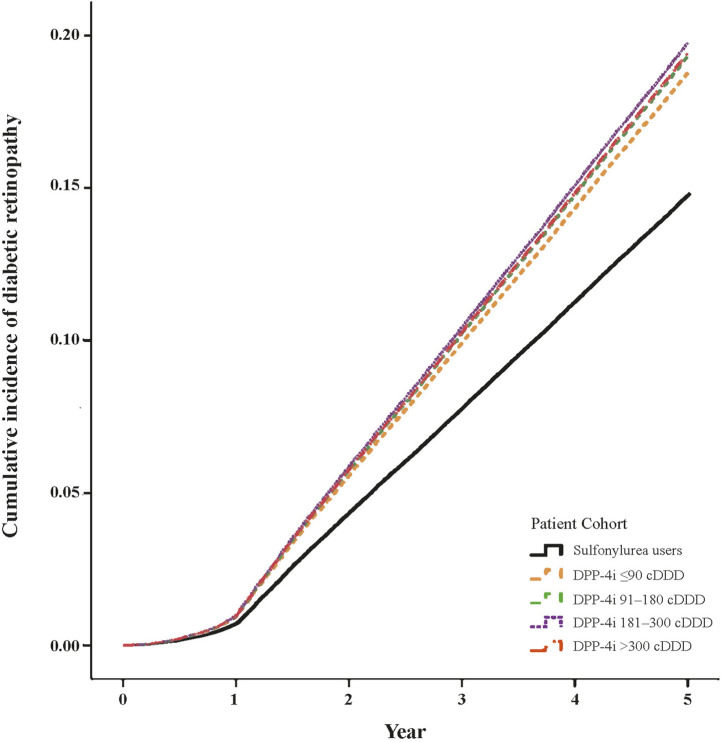
Cumulative incidence curves of diabetic retinopathy in DPP-4i users (log-rank test, P < 0.001).

Table 2 presents the risk of DR over the 5-year follow-up. After adjustment for relevant variables, we discovered that compared with the patients with sulfonylurea use, the patients with DPP-4i use at ≤90, 91–180, 181–300, and >300 cDDD had HRs of 1.43 (95% CI: 1.38–1.49), 1.66 (95% CI: 1.59–1.74), 1.82 (95% CI: 1.74–1.90), and 1.03 (95% CI: 1.01–1.06) for DR development, respectively. Figure 2 presents the cumulative incidence curves of DR and reveals a significantly higher incidence in the patients with DPP-4i use than in the patients with sulfonylurea use (log-rank test, *P* < 0.001). In addition, male patients exhibited a significantly lower risk of DR relative to female patients (HR: 0.88, 95% CI: 0.86–0.90), and the risk of DR increased with age. Regarding comorbidities, patients with obesity (HR: 0.53, 95% CI: 0.36–0.79) or hyperlipidemia (HR: 0.92, 95% CI: 0.86–0.99) had lower risks of DR relative to those without these comorbidities. Regarding other anti-diabetic agent use, patients who took anti-diabetic agents (meglitinides, metformin, α-glucosidase inhibitor, TZD, or insulin) had a higher risk of developing DR than those who did not take these medications.

**TABLE 2:** Risk of incident diabetic retinopathy in 5 years follow up.

Variables	Events	%	Unadjusted model		Adjusted model	
			HR (95% CI)	p-value	HR (95% CI)	p-value
Total	36,093	14.41				
cDDD of DPP-4i use						
Sulfonylurea users	22,172	13.28	Reference		Reference	
≤90	3,376	17.21	1.46 (1.41–1.51)	<0.001	1.43 (1.38–1.49)	<0.001
91–180	2,166	20.09	1.71 (1.63–1.78)	<0.001	1.66 (1.59–1.74)	<0.001
181–300	2,332	21.98	1.86 (1.78–1.94)	<0.001	1.82 (1.74–1.90)	<0.001
>300	6,047	14.23	1.05 (1.03–1.09)	<0.001	1.03 (1.01–1.06)	0.042
Sex						
Female	16,369	15.75	Reference		Reference	
Male	19,724	13.46	0.85 (0.84–0.87)	<0.001	0.88 (0.86–0.90)	<0.001
Age (year)						
20–49	10,275	12.03	Reference		Reference	
50–54	5,923	14.81	1.26 (1.22–1.3)	<0.001	1.28 (1.24–1.32)	<0.001
55–59	6,580	16.01	1.38 (1.34–1.43)	<0.001	1.41 (1.36–1.45)	<0.001
66–64	5,764	17.30	1.52 (1.47–1.57)	<0.001	1.55 (1.50–1.61)	<0.001
≥65	7,551	14.90	1.41 (1.37–1.45)	<0.001	1.49 (1.44–1.54)	<0.001
Income level (NTD)^a^						
≤19,200	8,466	14.36	Reference		Reference	
19,201–22,800	11,889	14.28	0.99 (0.96–1.01)	0.3248	0.98 (0.95–1.01)	0.206
22,801–36,300	6,782	14.69	0.99 (0.96–1.02)	0.5658	1.01 (0.97–1.04)	0.769
≥36,301	8,956	14.42	0.97 (0.94–0.99)	0.0241	0.99 (0.96–1.02)	0.690
Urbanization						
Level 1	9,886	14.55	Reference		Reference	
Level 2	11,478	14.61	1.01 (0.99–1.04)	0.3291	1.01 (0.98–1.03)	0.689
Level 3	6,413	14.65	1.03 (0.99–1.06)	0.1273	1.01 (0.98–1.05)	0.416
Level 4	4,824	13.95	0.98 (0.95–1.02)	0.333	0.96 (0.92–0.99)	0.014
Level 5	681	13.42	0.97 (0.9–1.05)	0.4718	0.91 (0.84–0.99)	0.023
Level 6	1,456	14.09	1.01 (0.95–1.06)	0.8809	0.95 (0.90–1.01)	0.091
Level 7	1,355	13.22	0.94 (0.89–0.99)	0.0313	0.90 (0.85–0.95)	<0.001
DCSI^a^						
0	25,103	14.17	Reference		Reference	
1	6,397	15.85	1.14 (1.11–1.17)	<0.001	1.13 (1.09–1.16)	<0.001
2	3,032	14.47	1.09 (1.05–1.13)	<0.001	1.07 (1.03–1.12)	<0.001
≥3	1,561	13.02	1.08 (1.02–1.13)	0.005	1.06 (1.01–1.12)	0.049
Comorbidities						
Obesity						
No	36,068	14.42	Reference		Reference	
Yes	25	7.91	0.51 (0.34–0.75)	<0.001	0.53 (0.36–0.79)	0.002
Anxiety						
No	36,044	14.41	Reference		Reference	
Yes	49	13.69	1.06 (0.80–1.40)	0.7074	0.97 (0.73–1.29)	0.827
Depression						
No	36,026	14.40	Reference		Reference	
Yes	67	17.36	1.35 (1.06–1.71)	0.0147	1.27 (0.99–1.61)	0.057
Hypothyroidism						
No	36,068	14.41	Reference		Reference	
Yes	25	16.23	1.43 (0.96–2.11)	0.0757	1.25 (0.85–1.85)	0.262
Hyperthyroidism						
No	36,061	14.41	Reference		Reference	
Yes	32	13.62	1.00 (0.71–1.41)	1	0.92 (0.65–1.30)	0.629
Migraine						
No	36,080	14.41	Reference		Reference	
Yes	13	16.46	1.18 (0.69–2.02)	0.5554	1.13 (0.65–1.94)	0.667
CKD^a^						
No	36,018	14.42	Reference		Reference	
Yes	75	11.40	1.10 (0.88–1.38)	0.405	0.96 (0.77–1.21)	0.735
Hypertension						
No	33,868	14.47	Reference		Reference	
Yes	2,225	13.53	1.04 (0.99–1.09)	0.0537	0.95 (0.91–0.99)	0.028
Hyperlipidemia						
No	35,146	14.43	Reference		Reference	
Yes	947	13.59	0.96 (0.90–1.02)	0.2195	0.92 (0.86–0.99)	0.017
Other anti-diabetic agents						
Meglitinides						
No	34,002	14.30	Reference		Reference	
Yes	2,091	16.41	1.25 (1.20–1.31)	<0.001	1.12 (1.07–1.18)	<0.001
Metformin						
No	5,204	12.21	Reference		Reference	
Yes	30,889	14.86	1.17 (1.14–1.21)	<0.001	1.24 (1.20–1.27)	<0.001
α-glucosidase inhibitors						
No	31,744	14.15	Reference		Reference	
Yes	4,349	16.63	1.22 (1.18–1.26)	<0.001	1.14 (1.11–1.18)	<0.001
TZD^a^						
No	32,405	14.16	Reference		Reference	
Yes	3,688	17.07	1.23 (1.19–1.27)	<0.001	1.18 (1.14–1.22)	<0.001
Insulins						
No	32,829	14.16	Reference		Reference	
Yes	3,264	17.54	1.42 (1.37–1.47)	<0.001	1.35 (1.30–1.40)	<0.001

^a^ Abbreviations: NTD, New Taiwan dollars (1 NTD ≒0.03 USD); DCSI, diabetes complications severity index; CKD, chronic kidney disease; TZD, thiazolidinediones.

Table 3 presents the risk of DR in patients treated with different types of DPP-4i. After adjustment for related variables, the results revealed that the patients using sitagliptin had a higher risk of DR than the patients using a sulfonylurea. Of the patients using linagliptin, those treated with ≤90 cDDD (HR: 1.07, 95% CI: 1.01–1.14), and 181–300 cDDD (HR: 1.16, 95% CI: 1.07–1.26) had a higher risk of developing DR, whereas those treated with >300 cDDD had a lower risk of DR. Of the patients using saxagliptin, those treated with ≤90 cDDD (HR: 1.14, 95% CI: 1.06–1.21), 91–180 cDDD (HR: 1.21, 95% CI: 1.11–1.33) and 181–300 cDDD (HR: 1.13, 95% CI: 1.02–1.25) had a higher risk of DR, whereas those treated with >300 cDDD had a lower risk of DR.

**TABLE 3:** Different DPP-4i and risk of incident diabetic retinopathy.

Variables	Events	%	Unadjusted model		Adjusted model	
			HR (95% CI)	p-value	HR (95% CI)	p-value
cDDD of DPP-4i						
Linagliptin						
≤90	1,050	15.39	1.10 (1.04–1.17)	0.002	1.07 (1.01–1.14)	0.032
91–180	525	14.32	1.01 (0.93–1.10)	0.848	0.99 (0.90–1.08)	0.745
181–300	554	16.79	1.19 (1.10–1.30)	<0.001	1.16 (1.07–1.26)	<0.001
>300	1,199	12.15	0.81 (0.76–0.85)	<0.001	0.79 (0.74–0.83)	<0.001
Saxagliptin						
≤90	922	16.30	1.17 (1.10–1.25)	<0.001	1.14 (1.06–1.21)	<0.001
91–180	479	17.46	1.25 (1.14–1.37)	<0.001	1.21 (1.11–1.33)	<0.001
181–300	380	16.69	1.17 (1.06–1.3)	0.002	1.13 (1.02–1.25)	0.020
>300	755	12.94	0.86 (0.80–0.93)	<0.001	0.83 (0.78–0.90)	<0.001
Sitagliptin						
≤90	2,400	16.32	1.28 (1.23–1.34)	<0.001	1.24 (1.19–1.29)	<0.001
91–180	1,417	19.21	1.52 (1.44–1.60)	<0.001	1.46 (1.38–1.54)	<0.001
181–300	1,391	20.80	1.64 (1.56–1.74)	<0.001	1.59 (1.51–1.68)	<0.001
>300	3,357	14.85	1.05 (1.02–1.09)	0.005	1.02 (0.99–1.06)	0.264
Vildagliptin						
≤90	1,448	16.37	1.17 (1.11–1.24)	<0.001	1.14 (1.08–1.20)	<0.001
91–180	563	16.58	1.17 (1.07–1.27)	<0.001	1.14 (1.05–1.23)	0.003
181–300	417	16.01	1.11 (1.01–1.22)	0.035	1.09 (0.99–1.20)	0.072
>300	563	12.07	0.79 (0.73–0.86)	<0.001	0.78 (0.72–0.85)	<0.001

Table 4 presents the results of subgroup analyses stratified by key comorbidities and concomitant antidiabetic medications, comparing the risk of DR in DPP-4i users versus sulfonylurea users. Among patients with specific comorbidities, DPP-4i use was associated with a significantly increased risk of DR in those with hypertension (HR: 1.30, 95% CI: 1.19–1.41) and hyperlipidemia (HR: 1.33, 95% CI: 1.17–1.52). A notable increase in DR risk was also observed in patients with migraine (HR: 10.24, 95% CI: 1.47–71.64), although the sample size was small, leading to wide confidence intervals. Regarding concomitant antidiabetic medications, DPP-4i use consistently showed a higher risk of DR across all strata, including patients using meglitinides (HR: 1.32, 95% CI: 1.21–1.44), metformin (HR: 1.30, 95% CI: 1.28–1.34), α-glucosidase inhibitors (HR: 1.30, 95% CI: 1.22–1.38), TZD (HR: 1.34, 95% CI: 1.26–1.43), and insulins (HR: 1.35, 95% CI: 1.26–1.44).

**TABLE 4:** The stratified analysis by different comorbidities and concomitant antidiabetic medications.

Variables	Risk of incident diabetic retinopathy (DPP-4i vs. Sulfonylurea [ref.])			
	Unadjusted model		Adjusted model	
	HR (95% CI)	p-value	HR (95% CI)	p-value
In patients with different comorbidities				
Obesity	1.01 (0.46–2.20)	0.992	0.91 (0.36–2.28)	0.840
Anxiety	1.33 (0.76–2.33)	0.317	1.34 (0.73–2.47)	0.341
Depression	1.23 (0.76–1.99)	0.390	1.55 (0.91–2.62)	0.105
Hypothyroidism	1.32 (0.60–2.90)	0.484	1.47 (0.46–4.69)	0.517
Hyperthyroidism	0.56 (0.27–1.17)	0.122	0.45 (0.20–1.03)	0.060
Migraine	3.23 (0.99–10.49)	0.051	10.24 (1.47–71.64)	0.019
Chronic kidney disease	1.18 (0.74–1.88)	0.489	1.09 (0.66–1.79)	0.746
Hypertension	1.37 (1.26–1.49)	<0.001	1.30 (1.19–1.41)	<0.001
Hyperlipidemia	1.41 (1.24–1.60)	<0.001	1.33 (1.17–1.52)	<0.001
In patients with different antidiabetic medications				
Meglitinides	1.31 (1.20–1.43)	<0.001	1.32 (1.21–1.44)	<0.001
Metformin	1.30 (1.27–1.33)	<0.001	1.30 (1.28–1.34)	<0.001
α-glucosidase inhibitors	1.31 (1.23–1.38)	<0.001	1.30 (1.22–1.38)	<0.001
TZD	1.35 (1.27–1.44)	<0.001	1.34 (1.26–1.43)	<0.001
Insulins	1.37 (1.28–1.47)	<0.001	1.35 (1.26–1.44)	<0.001

## Discussion

In our large-scale population-based retrospective cohort study, we discovered that patients with DM who were prescribed a DPP-4i at ≤90, 91–180, 181–300 and >300 cDDD had a higher risk of DR over the 5-year follow-up. Sitagliptin at cDDD 181–300 was associated with the highest DR risk. The current findings also demonstrated that female patients with DM receiving a DPP-4i had an increased risk of DR.

SDF-1 may also play a crucial role in the development of retinopathy; retinopathy development begins with damage to small blood vessels in the eye and progresses due to a neovascular response that is exacerbated by SDF-1 (Butler et al., 2005; Vidakovic et al., 2015). Elevated levels of SDF-1 have been found in the vitreous of the eyes in patients with ischemic ocular diseases, including proliferative diabetic retinopathy and retinopathy of prematurity (Butler et al., 2005; Sonmez et al., 2008). SDF-1α may worsen DR through its induction of angiogenesis and increased vascular permeability, which are central to the pathophysiology of DR. One study demonstrated that the serum SDF-1 level is closely related to hyperglycemia, hypercoagulability, and inflammation in patients with DM (Lu et al., 2021). Moreover, the SDF-1 level in the vitreous humor was found to be higher in patients with RD than in patients without RD. SDF-1 level is correlated positively with the duration and extent of RD (Otsuka et al., 2010).

In one study, sitagliptin prevented nitrosative stress, inflammation, and apoptosis in retinal cells and had beneficial effects on the integrity of the blood–retinal barrier in ZDF rat retinas (Goncalves et al., 2012). The study also suggested that sitagliptin alleviated the bovine retinal endothelial dysfunction caused by inflammation. Gonçalves et al. also discovered that sitagliptin exerted antioxidative effects on the retinas of rats (Goncalves et al., 2018). Moreover, linagliptin exerted protective effects on the microvasculature of the diabetic retina, probably because of its combined neuroprotective and antioxidative effects on the neurovascular unit (Dietrich et al., 2016). Another study found that linagliptin exhibited antiangiogenic effects in mice with oxygen-induced retinopathy (Kolibabka et al., 2018). The topical administration of DPP-4is was found to effectively prevent neurodegeneration and vascular leakage in the diabetic retina. Although this effect was likely due to an enhanced GLP-1 level, other mechanisms other than the prevention of GLP-1 degradation may also have played a role (Hernandez et al., 2017). However, an experiment revealed that long-term inhibition of DPP-4 destabilized the blood–retina barrier, potentially leading to retinal edema (Jackle et al., 2020).

In our study, the comparison group consists of patients treated with sulfonylureas. Sulfonylureas might be associated with an increased risk of DR compared to placebo. A network meta-analysis of 36 clinical trials revealed that sulfonylureas were associated with a higher risk of DR complications compared to a placebo, with an odds ratio (OR) of 1.67 (95% CI, 1.01–2.76) (Tang et al., 2018). In the present large-scale *population-based* study, we found that patients with DM using a DPP-4i at ≤90, 91–180, 181–300, and >300 cDDD had a higher risk of DR over the 5-year follow-up period. The effect of DPP-4is on DR risk varies, irrespective of whether a DPP-4is is used over the short or long term.

One study demonstrated that short-term (6-week) treatment with saxagliptin led to a decrease in retinal capillary flow in the microcirculation and a reduction in central systolic pressure in patients with DM (Ott et al., 2014). A small-scale retrospective observational study involving 82 patients with DM found that DPP-4i use was associated with reduced DR progression (Chung et al., 2016). Additionally, a retrospective cohort study obtained data from the electronic medical records of German patients (N = 630) and applied propensity score matching; the results revealed that vildagliptin treatment was associated with a lower incidence of retinopathy in clinical settings compared with sulfonylurea treatment (Kolaczynski et al., 2016).

Although these short-term and small-scale studies have provided positive findings, some studies have indicated that DPP-4is may result in adverse retinal outcomes. In the Trial Evaluating Cardiovascular Outcomes with Sitagliptin, patients receiving add-on sitagliptin therapy had a higher incidence of DR than those who did not receive the therapy (2.8% versus 2.2%) (Green et al., 2015). Trials and a meta-analysis have revealed that DPP-4is are associated with increased retinopathy risk (Kim et al., 2018; Kang et al., 2021). A study in a representative sample of the South Korean population also indicated that relative to other oral glucose-lowering agents, DPP-4is did not result in a higher overall risk of DR. However, the use of DPP-4is may be linked to an increased risk of retinopathy in the early treatment phase (<12 months) (Kim et al., 2018). A population-based cohort study found that patients with DM using a DPP-4i had higher risks of vitreous hemorrhage and macular edema than those who did not use a DPP-4i (Kang et al., 2021). The study indicated that add-on DPP-4i therapy may be associated with the progression of preexisting DR in patients with DM aged 40 years (Kang et al., 2021). A pairwise meta-analysis of RCTs indicated that DPP-4i use is associated with an increased risk of DR (OR: 1.27, 95% CI: 1.05–1.53) (Tang et al., 2018). Another meta-analysis indicated that DPP-4is slightly increased the risk of DR (relative risk: 1.17, 95% CI: 0.99–1.39), but the increase was nonsignificant (Tan et al., 2023). A study including a cohort representative of the US population in the 65-year age group found that DPP-4i use had a neutral effect on DR risk (Wang et al., 2018). Evidence from a meta-analysis of real-world studies indicates that DPP-4is may not significantly affect the incidence or progression of DR in patients with type 2 DM (Wang et al., 2024). The current population-based study revealed that patients with DM who used a DPP-4i had an increased risk of DR and that this risk increased in a dose-dependent manner over the 5-year follow-up period. The effect of DPP-4i on microvascular complications has shown inconsistent outcomes, with limited research specifically focusing on their impact on DR (Taylor and Lam, 2020). Results from several previous studies investigating the association between DPP-4i therapy and the risk of DR in individuals with DM have been inconsistent and inconclusive. However, as our data is derived from Taiwan’s NHIRD, which reflects a relatively homogeneous population, variations in findings may occur across different races and ethnicities. Well-designed and large-scale studies focusing on DR outcomes are needed be conducted to clarify the benefits and risks of DPP-4i therapy for DR in patients with DM.

In our study, we found that sitagliptin at cDDD 181–300 was associated with the highest risk of DR, followed by sitagliptin at cDDD 91–180. A study reported that after 4-week treatment, patients with DM treated with sitagliptin exhibited significant increases in the number of circulating endothelial progenitor cells (EPCs) and level of SDF-1α compared with controls (Fadini et al., 2010). This increase in the number of EPCs is probably mediated by the SDF-1α/CXCR4 pathway through DPP-4 inhibition, which prevents the breakdown of SDF-1α (Fadini et al., 2010). Lovshin et al. reported that sitagliptin significantly increased the plasma levels of the intact forms of SDF-1α and decreased the plasma levels of the truncated forms of SDF-1α in plasma (Lovshin et al., 2017). Similarly, 26-week treatment with linagliptin significantly increased the SDF-1α level of patients with DM, where placebo was administered in the control group (de Boer et al., 2020). Theoretically, linagliptin may exert adverse effects on the neurovascular unit because DPP-4 inhibition may promote the development of proliferative retinopathy. DPP-4 plays a crucial role in inactivating proangiogenic factors such as SDF-1α and HMGB-1 (Dietrich et al., 2016). We observed that linagliptin, saxagliptin and vildagliptin at cDDD >300 were associated with a lower risk of DR, whereas sitagliptin at cDDD >300 showed no such association. However, the mechanisms connecting cumulative DPP-4i dosage to the risk of DR remain unclear, further exploration through large-scale studies to evaluate the potential risks of DPP-4i therapy for DR in patients with DM.

SDF-1 is critically involved in the development of proliferative retinopathy (Butler et al., 2005). DPP-4is prevent the degradation of SDF-1α, thus increasing its active concentration (Lambeir et al., 2001; Fujita et al., 2014). The increase in SDF-1 caused by DPP-4is may exert adverse effects due to this protein’s neovascular effects; these adverse effects include proliferation and damage that are similar to the pathological processes involved in the development of DRR (Butler et al., 2005). Interestingly, SDF-1 inhibition affects the detached retina but not the normal retina; this finding indicates that the detached retina is more sensitive to environmental changes than is the normal retina. This tendency was also observed in retinal detachment (RD) in IL-6^−/−^ mice (Chong et al., 2008).

DPP-4is increase the accumulation of SDF-1α, which induces vascular leakage and angiogenesis in DR through the SDF-1α/CXCR4/Src/VE-cadherin signaling pathway (Lee et al., 2016). Activation of this pathway may disrupt the formation of the VE–cadherin–catenin complex, which plays a critical role in maintaining the integrity of endothelial cell–cell junctions (Monaghan-Benson and Burridge, 2009; Potter et al., 2005). Lee et al. indicated that DPP-4is caused disruptions in endothelial cell–cell junctions by triggering the accumulation of SDF-1α and the phosphorylation of vascular endothelial cadherin and that DPP-4is further increased retinal vascular permeability (Lee et al., 2016). Further larger and longer-term studies are warranted to determine SDF-1α levels after treatment with different types of DPP-4i.

DPP-4i use was consistently linked to an increased risk of DR across all subgroups, including patients receiving meglitinides, metformin, α-glucosidase inhibitors, TZDs, and insulin. However, the stratified analysis revealed variations based on different comorbidities. DPP-4i use was significantly linked to a higher risk of DR among patients with hypertension, hyperlipidemia, and migraine. Notably, the risk increase was particularly evident in those with migraine, although the small sample size resulted in wide confidence intervals.

The current study demonstrated that female patients with DM receiving a DPP-4i exhibited an increased DR risk. Increasing evidence indicates that sex may be a significant risk factor for DR (Nittala et al., 2014; Kajiwara et al., 2014; Ozawa et al., 2015). Some studies have indicated that female sex is an independent risk factor for the incidence and progression of overall DR and proliferative DR (Kajiwara et al., 2014; Awa et al., 2012). Among patients with DM and at least a 10-year history of DM who were aged >60 years, the prevalence of DR was higher among women than among men (Li et al., 2020). A study from Germany and Australia indicates that females are more likely to develop DR compared to males (Awa et al., 2012). Similarly, a study from Japan found that female patients with DM had a significantly higher prevalence of proliferative DR at baseline. The study also identified female gender as an independent risk factor for the development of DR, with females being more susceptible to visual impairment compared to males (Hashemi et al., 2017). The prevalence of DR is increasing among the elderly, and it is becoming a growing cause of vision loss in this elderly population (Leley et al., 2021). A study from Iran revealed that the prevalence of DR increased with age between 55 and 74 years. In the 55–59 age group, the prevalence was approximately 1.0%, rising progressively to a peak of 8.2% in the 70–74 age group. However, this trend was not observed in individuals aged 75 and older, where the prevalence dropped to 3.4% (Hashemi et al., 2017).

The major strength of the present study is the population-based design. By selecting study subjects from the entire population of Taiwan, our sample is highly representative and sufficient, thus mitigating selection bias. Additionally, the study employed a long follow-up (5 years) after the initiation of DPP-4i treatment. This long follow-up led to findings with sufficient statistical power regarding the relationship between DPP-4i use and DR risk.

This study has several limitations. First, we could not collect data on family history of DR for our patients. Additionally, we could not collect information on lifestyle characteristics relevant to the risk of DR, such as smoking, alcohol consumption, glycated hemoglobin level, body mass index, physical activity, personal history, and dietary habits, which are potential confounding factors. Second, our diagnoses of DR and other comorbidities were entirely dependent on *ICD-9-CM* and *ICD-10-CM* codes. Nonetheless, to verify the accuracy of diagnoses, the National Health Insurance Bureau of Taiwan randomly reviews patient charts and conducts patient interviews. Third, the NHIRD does not contain laboratory records, including serum *levels of* SDF-1, which limited the current study. Furthermore, this study did not include an ophthalmological examination of diabetic patients to confirm a DR diagnosis. As a result, the potential underdiagnosis of DR may influence the study’s findings. Fourth, we were uncertain whether all patients adhered to their prescribed antidiabetic medications. As per the stipulations of the National Health Insurance program of Taiwan, physicians may prescribe antidiabetic medications to patients even if their symptoms are mild and a pharmacological intervention is not yet required to reduce a patient’s blood sugar level. Although some patients may have had mild symptoms that could be managed through diet or exercise alone, we were unable to identify such patients, which is a limitation of our study. Finally, this study was an epidemiological study. Although many risk factors for DR were controlled for in the analysis to confirm the correlation between DPP-4i use and DR risk, numerous potential risk factors could not be included. Consequently, a causal relationship could not be established in this study.

## Conclusion

In summary, in this study, patients with DM who received a DPP-4i at ≤90, 91–180, 181–300 and >300 cDDD over the 5-year follow-up period. Sitagliptin at cDDD 181–300 was associated with the highest risk of DR. Therefore, when selecting anti-diabetic treatments for patients with DM and preexisting DR, the potential of DPP-4is to accelerate DR progression should be considered.

## Data Availability

The data analyzed in this study is subject to the following licenses/restrictions: Data in this study was retrieved from Taiwan’s NHIRD. All data were administrated by the Taiwan National Health Insurance (NHI) Bureau. Because of the Personal Information Protection Act and related regulations, data sets are not publicly available. Requests to access these datasets should be directed to Requests to access the data can formally be submitted to the NHI Bureau (https://dep.mohw.gov.tw/DOS/cp-5119-59201-113.html).
